# A survey of current surgical treatment of early stage breast cancer in China

**DOI:** 10.18632/oncoscience.445

**Published:** 2018-08-22

**Authors:** Xin Zhang, Ying Wang

**Affiliations:** ^1^ Department of Radiation Oncology, Chongqing University Cancer Hospital, Chongqing Cancer Institute, Chongqing Cancer Hospital, Chongqing, People’s Republic of China

**Keywords:** beast cancer, China, current treatment, questionnaire survey

## Abstract

The purpose of this national survey is to explore the patterns of surgical management for early stage breast cancer patients in China. A paper questionnaire survey was sent to the representatives from 520 hospitals who attended an international symposium in Guangzhou, China, 2014. The questionnaire included demographic information, initials and most preferred approaches for breast cancer surgery. The results were presented descriptively. The response rate was 42.5%. Only 7% of hospitals with >50% rate of breast conserving surgery (BCS). Intraoperative frozen sections and additional cavity margins assessment were used at 88% and 30.9% of hospitals, respectively. For invasive carcinoma, 15% of participants defined an adequate margin as no tumor cells on the ink. Sentinel lymph node biopsy (SLNB) was routinely performed in 93.2% of hospitals. Only 16.6% of hospitals would embrace the conclusions of the American College of Surgeons Oncology Group (ACOSOG) Z0011 study and omit axillary lymph node dissection (ALND) for patients who fit the Z0011 criteria. The current patterns for the management of breast cancer patients are still lagging behind. Chinese doctors need to catch up with the updated results of the cutting-edge clinical studies and multiple measures are in need to improve this situation.

## INTRODUCTION

Breast cancer is the most commonly diagnosed cancer and the sixth leading cause of cancer death in Chinese women in 2015 [1]. Increasing numbers of early stage breast cancer patients were detected [2]. A multicenter nationwide study showed that clinical stage I and II breast cancer accounted for 60.6% of cases in China [3].

Early stage breast cancer management is multidisciplinary in nature, including surgery, radiotherapy and systemic therapy. However, the role of breast surgeons in diagnosis and local oncological control remains significant. It would be informative to know the current surgical treatment status of early stage breast cancer in China. There were several studies that focus on the epidemiology or the current treatment of breast cancer. The patients’ information was collected from one province, a nationwide multi-center analysis or an online database [3-5]. However, no detailed information came from a survey of surgeons. The treatment methods received by the Chinese patients are usually dependent on the surgeons’ decision making. The surgical practice, attitudes and decision making towards breast cancer surgery may vary among breast surgeons owing to the experiences of the surgeons or the limited resources in some institutions. Therefore, a survey from the surgeons can reflect the current surgical treatment status of early stage breast cancer in China.

The surgical treatment methods for early stage breast cancer are developing quickly, and controversy issues still impact the doctors’ decision making and attitudes. There were several surveys sent to members of the medical society regarding controversial issues, such as definition of an adequate margin [6-9]. However, little is known regarding the Chinese breast surgeons’ attitudes about controversial issues or whether they grasp the new clinical studies.

Therefore, we conducted a national survey by a self-reported written questionnaire that was sent to the participants of a national breast symposium held in Guangzhou city. The purpose of this national survey is to explore the patterns of surgical management for early stage breast cancer patients in China. We will also investigate whether Chinese surgeons can catch up with the results of cutting-edge clinical studies.

## RESULTS

### Response rates and demographics

A total of 221 hospitals responded to the questionnaires, covering all of the mainland, except for Anhui province and the Tibet autonomous region. Of the respondents, a considerable number (36.2%) were from Guangdong province where the symposium was held. The response rate was 42.5% (221/520), and 86% of respondents completed the whole questionnaire, while 97% of respondents completed at least 70% of the questions.

Most respondents were senior professionals and came from academic-based (76.9%), “Grade A class 3” (80.5%) hospitals. About one half of the department type was breast center. 73.3% of respondents devoted more than half of their time to breast surgery (Table 1).

### Issues of preoperative pathological diagnosis

We observed that more than 90% of patients could acquire preoperative pathological diagnosis at only 26.2% of hospitals. Core needle biopsy was used at most hospitals for breast tumor of BIRADS 1-3 (32.1%) and BIRADS 4-5(53.8%). About one fourth of hospitals still chose diagnostic open excisional biopsy for both BIRADS 1-3 and BIRADS 4-5 breast tumor (Table 2).

### Issues of breast surgery

**Figure 1:**
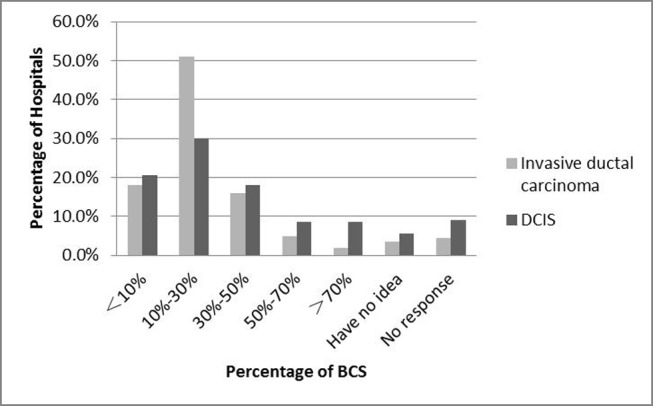
Percentage of BCS for primary invasive ductal carcinoma / DCIS at the surveyed hospitals

In our results, 9.5% (20/221) of hospitals surveyed never performed BCS. Their reasons were as follows: there was no person skilled at the surgical technique for BCS (n=3), refusal from patients (n=8), or the lack of radiotherapy department (n=5) or pathology department (n=2). Only 7% and 17% of hospitals had more than a 50% proportion of BCS for primary invasive ductal carcinoma and ductal carcinoma in situ (DCIS), respectively (Figure 1).

In our results, 97% (195/201) of hospitals used intraoperative techniques to assess the margin status when performing BCS. Additionally, 37.1% of hospitals assessed cavity margins, 31.4% used tumor specimen, and 30.9% used both methods. A majority of (87%) hospitals chose the intraoperative frozen section to identify the margin status, while a few chose imprint cytology (2%), gross inspection (3%) and ultrasound (1%).

**Figure 2:**
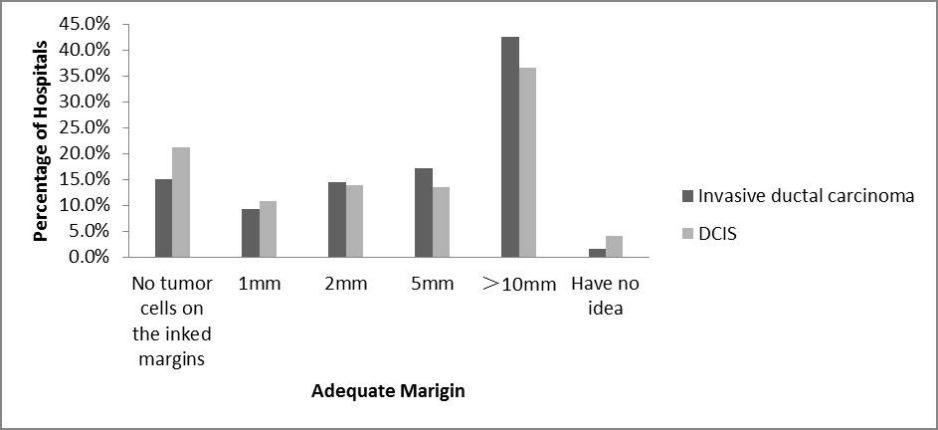
Definition of an adequate margin when performing BCS for invasive ductal carcinoma/DCIS

Additionally, participants were asked how to define an adequate margin when performing BCS (Figure 2). For invasive ductal carcinoma, only 15% considered an adequate margin with no tumor cells on the inked margins, while 42.5% required 10 mm of clear tissue, 9.3% required 1 mm, 14.4% required 2 mm and 17% required 5 mm. For DCIS, margins were considered adequate by 21.1% when there were no tumor cells on the inked margins.

**Figure 3:**
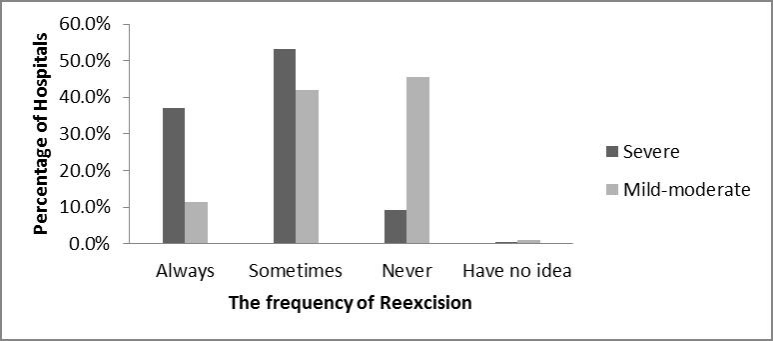
How frequently the hospitals perform of margins with severe/mild-moderate atypical hyperplasia

When we asked questions about re-excision with a positive intraoperative margin status, the majority of (90%) hospitals considered re-excision of the focal area where the positive margin was located, while 8% excised the whole cavity again. We also asked whether re-excision of the margins was necessary for atypical hyperplasia. 53.1% of the hospitals would “sometimes” recommend re-excision for severe atypical hyperplasia and 41.8% for mild-moderate atypical hyperplasia (figure 3). The hospitals surveyed were more likely “always” to recommend re-excision for severe atypical hyperplasia (37.1%) than mild–moderate (11.4%).

### Issues of SLNB

SLNB was reported to be routinely performed by 93.2% of the hospitals for patients with clinically negative axillary lymph nodes. The major reasons for abandoning SLNB were lack of resources or equipment (n=8), lack of accurate pathology diagnosis (n=6), or lack of skilled surgical technique (n=2). The majority (90.5%) of hospitals could use intraoperative frozen section to analyze the SLNs. When asked about the SLNB technique, 77.7% of hospitals reported that they use dye only, while the remaining 22.3% used both dye and radiotracer. Of those, methylene blue was the most commonly used dye (80.5%).

We asked participants when the immunohistochemical (IHC) stains of the SLNs should be conducted. A small number (4%) considered that IHC stains of SLNs were necessary when the hematoxylin and eosin (HE) stains were tumor free, while 16.6% would perform IHC stains regardless of the result of the HE stains, and 26.1% recommended it to identify the receptor status of the SLNs when there was a positive result by HE stains. 21.1% of hospitals never conducted IHC stains.

About one third of hospitals would perform percutaneous node biopsy before SLNB. Of those, 24.1% used a fine needle aspiration biopsy, while 9.9% preferred the core needle. When asked, “Does your department conduct ALND for clinical stage T1-2N0M0 breast cancer patients confirmed with 2 or less positive SLNs who received BCS followed by whole breast radiation and adjuvant systemic therapy”, a significant proportion (80%) of the hospitals answered “Yes”. In addition, in a half of hospitals surveyed, SLNB was routinely carried out in breast cancer patients with positive nodes but negative axillary conversion after NACT. 48% of hospitals carried out SLNB after NACT for clinical axillary negative patients, while 43% did the opposite.

## DISCUSSION

Our study is the first to report the current surgical treatment status of early stage breast cancer in China based on a questionnaire sent to surgeons during a national symposium. The response rate was 42.5%, which is similar to other surveys in the west [6,8,9]. Most respondents came from highly ranked Chinese’s hospitals. Two thirds of participants were senior professionals and half worked in specialized breast cancer centers. We inferred that the participant surgeons know very well about the current surgical treatment status of early stage breast cancer in their departments. Therefore, the results of the survey responded by the surgeons could reflect the hospitals.

### Issues of preoperative pathological diagnosis

The European Society of Breast Cancer Specialists (EUSOMA) defined a quality indicator for breast centers, stating that more than 90% of patients had a preoperative definitive diagnosis [10]. However, in our survey, only 27% of hospitals reached this standard. A preoperative core needle biopsy is the gold standard method to diagnose primary breast cancer, [11,12] and was endorsed by only half of respondents for BIRADS 4-5 breast tumor in this survey. A study in Beijing also showed that only 34.1% of the breast cancer patients were diagnosed by a core needle biopsy. However, approximately one fourth of hospitals still chose diagnostic open excisional biopsy frequently for breast tumors, compared with 15%-20% of Canadian and American surgeons in a survey [6]. Excisional biopsy will result in unnecessary surgical procedures and increased cost [13], as well as decreasing the accuracy of the sentinel node biopsy [14].

### Issues of breast surgery

BCS with adjuvant radiotherapy is a standard surgical procedure for early stage breast cancer. In this survey, only a small number of hospitals never developed BCS. However, the rate of BCS was still very low (7% of hospitals with >50% rate of BCS), which means that only 7% reached the higher proportion (64%) of BCS in the USA [15]. A nationwide survey in China also reported only 5.5% of BCS cases [3]. The reasons for the low frequency of BCS in China may be a lack of resources for radiotherapy and pathological supports, or the incomprehension of patients.

**Table 1:** Respondent Characteristics

Characteristic	n	%
Professional title of the participants		
Primary	18	8.1
Intermediate	52	23.5
Senior	151	68.4
Hospital level		
Grade A class 2	28	12.7
Grade B class 2	2	0.9
Grade A class 3	178	80.5
Grade B class 3	13	5.9
Practiced facility		
Academic	170	76.9
Non-academic	47	21.3
Private	4	1.8
Department type		
Breast cancer center	128	57.9
Non-breast cancer center	93	42.1
If you devoted more than half time to breast surgery		
Yes	162	73.3
No	59	26.7

Intraoperative margin assessment when performing BCS can reduce final positive margin rates. A frozen section was reported as having the highest sensitivity and specificity compared with imprint cytology or macroscopic assessment [16,17]. However, frozen sections increased the operating time from 20-30 min, which may result in additional costs [16]. Margin sampling can come from cavity margins or tumor specimens. Several studies reported that sending a separate cavity margin sample to pathology can help to more easily obtain a negative margin and minimize the local recurrence rates [18-20]. In this survey, almost all hospitals assessed the margin status intraoperatively, and the majority used intraoperative frozen sections. One third of the hospitals assessed both the cavity margins and tumor specimens. In contrast, many surgeons in USA and Canada never use intraoperative frozen sections, nor do they send additional cavities [6,7].

The definition of an adequate margin when performing BCS is a controversial issue. Several major randomized trials did not have a standard definition of the adequate margin. However, “no tumor cells on the inked margin” was considered a sufficient negative margin by the Society of Surgical Oncology (SSO) and the American Society of Radiation Oncology (ASTRO) in a consensus panel in 2013, which was based primarily on a systematic review and meta-analysis [21,22]. There existed several surveys about this topic in Western countries. “No tumor cells on the ink” was accepted by 40% of Canadian respondents [23], 27.6% of European respondents [9], and 15% of USA respondents [7]. In our survey, only 15% of respondents considered an adequate margin with no tumor cells on the inked margins for invasive ductal carcinoma. However, the USA respondents were surveyed in 2009 before the SSO and ASTRO consensus panel. So Chinese surgeons were more likely to support a wider margin.

### Issues of SLNB

SLNB is now considered a standard practice for breast cancer patients with clinically negative axillary lymph nodes. In our survey, 93.2% of hospitals routinely performed SLNB, compared with 87.8% in Latino America [24], 52.0% in United Kingdom [25], 88.9% in North America and 66.1% in Europe [26].

A meta-analysis showed that the dual technique (combined with dye and radiotracer) is the gold-standard for the successfully identification of SLNs [27], but dye alone was also reliable and accurate [28]. In our survey 22.3% of hospitals used the dual technique, compared with 64.5% in the UK [25]. Intraoperative assessment of SLNs allows immediate ALND in patients with positive SLNs, avoiding a second surgery. In our survey, intraoperative frozen section for SLNs analysis was used by the majority (90.5%) of hospitals, compared with 41.9% in Latin America and 2.6% in the UK [24,25].

**Table 2:** Preoperative pathological diagnosis

Approach	n	%
The percentage of patients who acquired preoperative pathological diagnosis		
<30%	40	18.1
30%—50%	39	17.6
50%—70%	41	18.6
70%—90%	33	14.9
>90%	58	26.2
Have no idea	10	4.6
The method of preoperative pathological diagnosis for breast tumor of BI-RADS 1-3		
Fine needle aspiration	15	6.7
Core needle biopsy	71	32.1
Minimally invasion excisional biopsy	63	28.5
Diagnostic open excisional biopsy	52	23.5
No preoperative pathological diagnosis	17	7.7
Have no idea	3	1.5
The method of preoperative pathological diagnosis for breast tumor of BI-RADS 4-5		
Fine needle aspiration	13	5.9
Core needle biopsy	119	53.8
Minimally invasion excisional biopsy	32	14.5
Diagnostic open excisional biopsy	51	23.1
No preoperative pathological diagnosis	4	1.8
Have no idea	2	0.9

IHC techniques were not recommended for routine use by neither NSABP B32 nor ACOSOG Z0010 [29,30]. It is possible that, IHC can be useful for confirming or excluding suspicious findings by HE stains. In our survey, about one fifth used IHC to detect SLNs, and one fourth used it to identify the receptor status of SLNs with positive results by HE stains.

ACOSOG Z0011 showed noninferiority for OS and DFS in breast cancer patients with SLNB alone compared with those undergoing SLNB plus ALND, the included patients were clinical stage T1-2N0M0, which was confirmed with 2 or less positive SLNs and received BCS followed by whole breast radiation and adjuvant systemic therapy [31]. Two studies in the USA reported the impact of the trial on the patterns in the surgeons’ practice. One reported that the rate of ALND was smaller after the trial presentation (84% to 63%; P<0.01) and publication (83% to 62%; P<0.01) [32]. Similarly, in another report, the rate of ALND decreased from 85% to 24% after the release of the trial [33]. However, we observed that 80% of hospitals still conducted ALND for patients who meet the Z0011 criteria.

Several studies reported that preoperative node sampling by needle biopsy can identify and triage patients with node metastases directly to ALND, avoiding an unnecessary SLNB procedure [34,35]. However in the ACOSOG Z0011 era [31], the value of preoperative node sampling is narrowed to the patients with a larger tumor or who will undergo NACT. In our study, approximately one third of hospitals still performed node biopsy before SLNB.

Even though NACT seems to impact the detection of SLNs and lower the accuracy of SLNB, some studies showed that SLNB can be offered before or after NACT with an acceptable false-negative rate (FNR) [36,37]. In our study, about half of hospitals would conduct SLNB before NACT. Nevertheless, the role of SLNB for patients with clinically positive nodes but negative axillary conversion after NACT remains controversial. The FNR for the situation was reported at 14.2% and 16.7% by two prospective studies [38,39]. However, there were no positive results in local axillary recurrence reported. Therefore, we have no idea whether the high FNR can result in a worse outcome because of high developed radiotherapy and systemic therapy. We also observed that the hospitals surveyed hold different opinions.

In general, we found that both overtreatment and undertreatment for patients with early stage breast cancer occurred. Preoperative diagnosis was insufficient and some participants still used excisional biopsy. Almost all hospitals can carry out BCS, but the proportion of BCS was considerable lower than the developed countries. Compared with the Western countries, Chinese surgeons have a more conservative attitude. Intraoperative frozen sections and additional cavity margins assessment during BCS were used more frequently in China, and Chinese surgeons were more likely to support a wider margin. When conducting SLND, Chinese surgeons preferred intraoperative frozen sections and IHC techniques. Furthermore, the recent topics seemed to have less impact on the practice of Chinese surgeons, such as ACOSOG Z0011. And Chinese surgeons also did not reach an agreement on the controversial issues, such as the sequence between SLNB and NACT.

Chinese doctors should pay more attention to the most updated guidelines or consensuses about the treatment of breast cancer to improve the outcome of patients in China.

### Limitations

There were several limitations in our study. First, the survey was based on a self-reported questionnaire, which might not reflect actual practice patterns. Second, one third of respondents were from Guangdong province where the symposium was held, and some province had only one responder. So, the differences between locations could not be analyzed and presented. Furthermore, almost all participants came from high level hospitals, and the status of some rural or community hospitals was unclear. Lastly, the questionnaire design could not cover the reasons of the chosen treatment option. And we need further study to explore the reasons.

## METHODS

### Survey methodology

The design of this study was an anonymous survey that was sent to the surgeons who attended the 5th International Oncoplastic Breast Surgery Symposium in Guangzhou, China from September 18 to September 22, 2014. Approximately 1000 participants from 520 hospitals attended the symposium. An initial paper version of the questionnaire was sent to a surgeon who was selected randomly as the representative of one hospital. The results of the survey responded by the surgeons could reflect the hospitals. One day later, an oral reminder was sent to the non-responders. At the end of the symposium, the completed questionnaires were collected.

### Questionnaire

The questionnaire was constructed by the authors, in consultation with professors from Breast Tumor Center at Sun Yat-sen Memorial Hospital. The survey was subsequently pilot tested with 10 surgeons from other hospitals in Guangdong province. They responded to the survey and gave feedback on it. The survey was revised according to their suggestions. Answers from the 10 surgeons for survey development were not included in this data set.

The questionnaire was divided into four sections. The first section was regarding demographic issues such as the hospital’s location and categories, as well as the department characteristic. The second section concerned preoperative pathological diagnosis. In the third section, participants were asked about the proportion of BCS, methods for intra-operative margin assessment, definition of adequate margin and extent of resection. The last section was about sentinel lymph node biopsy (SLNB), such as the methods to detect and analyze sentinel lymph nodes (SLNs), the recognition of Z0011 trial result, the sequence between SLNB and neoadjuvant chemotherapy (NACT).

### Statistical analysis

All data were coded and checked for errors by the principal investigator. Missing and ambiguous responses were excluded from the analysis. The results of surveys were entered into a database and analyzed by using SPSS (Version 19.0, IBM Crop, New York, USA). The results were classified by the percentage distribution and presented descriptively

## CONCLUSION

To the best of our knowledge, our study is the first to report the current treatment status of early stage breast cancer based on a questionnaire survey of hospitals. There were issues of both overtreatment and undertreatment for patients with early stage breast cancer. During BCS and SLND, Chinese surgeons kept a more conservative attitude. The current patterns for the management of breast cancer patients are still lagging behind. Chinese doctors need to catch up with the updated results of cutting-edge clinical studies and multiple measures are in need to improve this situation.

## Abbreviations

BCS: breast conserving surgery
SLNB: Sentinel lymph node biopsy
ACOSOG: American College of Surgeons Oncology Group
ALND: axillary lymph node dissection
SLNs: sentinel lymph nodes
NACT: neoadjuvant chemotherapy
DCIS: ductal carcinoma in situ
IHC: immunohistochemical
EUSOMA: European Society of Breast Cancer Specialists
SSO: Society of Surgical Oncology
ASTRO: the American Society of Radiation Oncology
FNR: false-negative rate
